# Supplementary material for: The effect of intrapartum prolonged oxygen exposure on fetal metabolic status: secondary analysis from a randomized controlled trial
Source: Front Endocrinol (Lausanne). 2023 Jun 27;14:1204956. doi: 10.3389/fendo.2023.1204956 (PMC10335765; doi:10.3389/fendo.2023.1204956)
Supplement: Supplementary file 3 [file DataSheet_3.zip › Result-X101SC21092977-Z01-J001-B1-42/4.MetDiffAnalysis/O.vs.A/O.vs.A_neg_corr.pdf]

O.vs.A

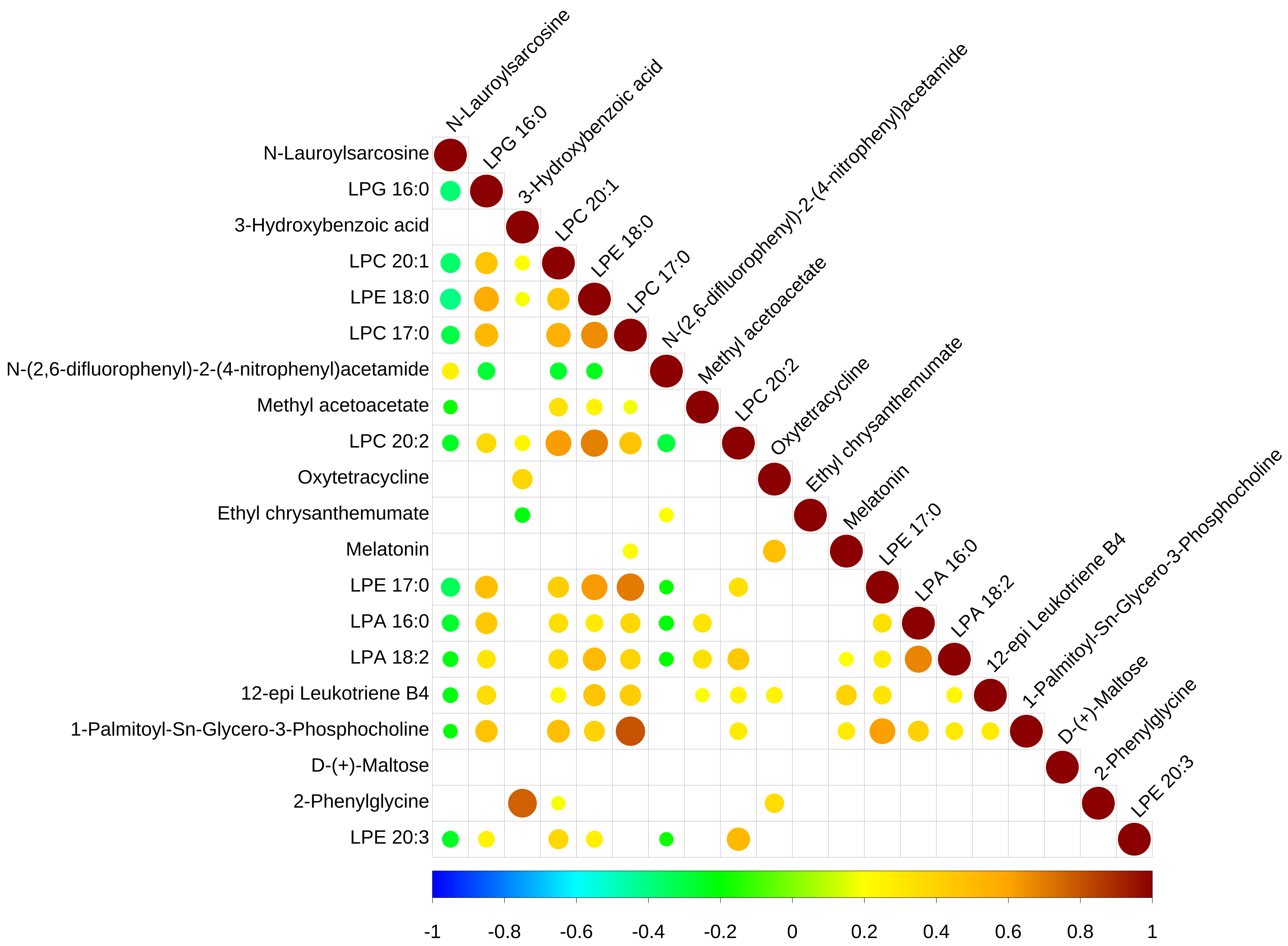

N-Lauroylsarcosine
LPG 16:0
3-Hydroxybenzoic acid
LPC 20:1
LPE 18:0
LPC 17:0
N-(2,6-difluorophenyl)-2-(4-nitrophenyl)acetamide
Methyl acetoacetate
LPC 20:2
Oxytetracycline
Ethyl chrysanthemumate
Melatonin
LPE 17:0
LPA 16:0
LPA 18:2
12-epi Leukotriene B4
1-Palmitoyl-Sn-Glycero-3-Phosphocholine
D-(+)-Maltose
2-Phenylglycine
LPE 20:3
-1
-0.8
-0.6
-0.4
-0.2
0
0.2
0.4
0.6
0.8
1
